# Adaptation and Preliminary Validation of the Fear of Coronavirus Vaccination Scale in the Prospective Study among a Representative Sample of Polish, Israeli, Slovenian, and German Adults during the COVID-19 Pandemic

**DOI:** 10.3390/ijerph191811587

**Published:** 2022-09-14

**Authors:** Dominika Ochnik, Aleksandra M. Rogowska, Joy Benatov, Ana Arzenšek

**Affiliations:** 1 Faculty of Medicine, University of Technology, 40-555 Katowice, Poland; 2 Institute of Psychology, University of Opole, 45-052 Opole, Poland; 3 Department of Special Education, University of Haifa, Haifa 3498838, Israel; 4 Faculty of Management, University of Primorska, 6101 Koper, Slovenia

**Keywords:** anxiety, COVID-19 pandemic, depression, fear of COVID-19, fear of vaccination, fear of coronavirus vaccination scale (FoCVVS), mental health, physical health, stress, vaccination status, well-being

## Abstract

Although concerns about harm and side effects are among the most important factors determining vaccine hesitancy, research on the fear of vaccination is sparse. The purpose of this study is a validation the Fear of Coronavirus Vaccination Scale (FoCVVS), adapted from the Fear of COVID-19 Scale. A representative sample of 1723 young adults aged 20–40 from Poland, Israel, Slovenia, and Germany participated during two time-points of the third COVID-19 pandemic wave. The online survey included demographic variables and several well-being dimensions, including gender, vaccination status, fear of coronavirus (FoCV-19S), physical health (GSRH), life satisfaction (SWLS), and perceived stress (PSS-10), anxiety (GAD-7), and depression (PHQ-9). Exploratory factor analysis (EFA) was performed at T1, and confirmatory analysis (CFA) at T2. The second-order two-factor structure demonstrated the best fit and very good discriminant and convergent validation. The general factor of the FoCVVS included two subscales assessing the emotional and physiological symptoms of fear of vaccination. Unvaccinated people showed higher levels of fear of vaccination than those vaccinated. A vaccination status, fear of vaccination T1, and fear of COVID-19 T1 were significant predictors of fear of vaccination T2. Vaccination-promoting programs should be focused on decreasing fear and enhancing the beneficial effects of vaccination.

## 1. Introduction

Vaccination decisions and hesitancy are essential to current research during the COVID-19 pandemic. Studies indicated that COVID-19 vaccines could decrease the severity of coronavirus infection, serious side effects, and death [1,2,3]. Despite the hard scientific evidence, many people, especially young adults, remain unvaccinated [4,5,6,7,8,9]. Vaccination prevalence seems to differ depending on geographic region or country [10,11]. For example, on 9 June 2021 (when the data were collected in the current study), there were 24–25% of fully vaccinated people in Germany, Poland, and Slovenia, whereas 56% in Israel [12]. The main barrier to vaccination is distrust in the effectiveness of vaccination and fear of vaccination side effects, including infertility, harm, severe disability, or even death [4,6,7,8,9,13,14,15,16,17,18,19]. 

Although fear of vaccination is one of the most important reasons for hesitancy, the study of fear of vaccination in hesitancy research is sparse. The main barrier to research seems to be a lack of reliable tools to measure the fear of vaccination. Previous studies have assessed fear, worry, or concerns about vaccination using self-developed single questions, which were never examined regarding reliability or validity e.g., [7,8,9,15,16,17,19]. Hamilton and Hagger [20] developed the Vaccination Concerns in COVID-19 Scale (VaCCS), but instead of fear, this tool measures beliefs and concerns about COVID-19 vaccines on 35 items included in eight subscales (socio-political, health beliefs and outcomes, trait measures, intentions to receive the COVID-19 vaccine). Similarly, Gregory et al. [21] developed a seven-point COVID-19 Vaccine Concern Scale (CVCS). However, still, this measure examines the cognitive aspects of the perceived reasons, attitudes, and beliefs associated with hesitancy rather than the symptoms of fear of vaccination. Recently, Kotta et al. [22] developed the Multidimensional COVID-19 Vaccine Hesitancy Scale (CoVaH), with three factors: skepticism, risk perception, and fear of the COVID-19 vaccine. The third factor assessed symptoms of fear of vaccination on a four-item scale, but the sensitivity of this subscale for screening unvaccinated people was weak (52.7%). Sato and Fintan [23] developed four questions about fear of needles, side effects of vaccines, concerns that the vaccine is of no benefit in preventing disease, and is harmful, which were rated on a four-item response scale. Although the scale is promising, the structure and parametric properties are unknown since the scale was never validated. Finally, Malas and Tolsá [24] adapted the Fear of COVID-19 Scale (FoCV-19S) to measure fear of vaccination in a sample of 2175 Spanish adults. However, the loading weight of item 5 (“When watching news and stories about COVID-19 vaccine on social media, you become nervous or anxious”) did not fall into sufficient criteria (factorial load less than 0.50, interpreted as fair), so item 5 was removed from the newly adapted Vaccination Fear Scale (VFS-6). In summary, all currently existing scales are not entirely suitable for measuring fear of vaccination.

In this study, we will adapt the original FoCV-19S developed by Ahorsu et al. [25], in a large representative sample of young adults from four countries: Germany, Israel, Poland, and Slovenia. We believe that the fear of COVID-19 vaccination is part of the fear of COVID-19 as a more general latent construct. We assume that both fears of COVID-19 and COVID-19 vaccination share the same symptoms of fear but differ in the source of fear (general COVID-19 versus COVID-19 vaccination). The reason to adopt the FoCV-19S for measurement of fear of coronavirus vaccination is that the FoCV-19S is an excellent standardized instrument to assess emotional and physiological symptoms of coronavirus fear. The FoCV-19S was developed based on several steps, starting from an extensive literature review, then the initial set of 30 items was evaluated and reduced by two expert panels and assessed using the pilot test and factor analysis [25]. The FoCV-19 was adapted and used in many countries worldwide during the COVID-19 pandemic, showing very good reliability and validity in various languages worldwide e.g., [26,27,28,29,30,31,32,33]. 

Fear symptoms are relatively easy to recognize and manage. A person who becomes aware of fear can take action to lower the level or eliminate the source of that fear. Therefore, it seems essential to measure both emotional and physiological fear of vaccination symptoms to engage in reducing them and, consequently, to increase vaccination in populations worldwide. It seems important during the global pandemic to find a universal tool to measure fear of vaccination, which will be valid in various cultures and independent of language. Such universal measures allow for the comparison of fear of vaccination globally, and they can speed up the development of appropriate prevention strategies against unnecessary coronavirus infections and deaths. Therefore, the present study will validate and compare the fear of vaccination scale across four languages: German, Hebrew, Polish, and Slovenian. German, Polish and Slovenian languages come from Europe, while Hebrew is from Asia. German and Polish languages and cultures differ substantially, but Germany and Poland border each other. Both Polish and Slovenian are Slavic languages, but Poland and Slovenia differ substantially in geographical regions of Europe. Hebrew is most distant from the other three languages and uses no Latin letters, but people from Israel share European culture.

Previous studies found one-factor [25,26,27,28], two-factor [29,30], and bi-factor [31] structures in the FoCV-19S, with two subscales related to the emotional and physiological responses to COVID-19. In this prospective study, we will examine one-factor and two-factor models using exploratory factor analysis (EFA) at the first time-point and using confirmatory factor analysis (CFA) during the second measurement. Country and gender heterogeneity will also be explored in our study to check whether the new FoCVVS is resistant to cultural differences.

To examine the concurrent validity of the FoCVVS, we will conduct a Student’s *t*-test regarding vaccination status and use correlation and regression analyses to investigate associations between the FoCVVS and well-being dimensions, including general self-rated health, stress, anxiety, depression, life satisfaction, and fear of COVID-19. Babicki et al. [34] reported higher stress levels, fear of COVID-19, and depression among unvaccinated young adults (aged between 18 and 29) than those vaccinated. Similarly, Alam et al. [35] found higher health problems (including general health, depression, and post-traumatic stress disorder) in unvaccinated than in vaccinated healthcare workers. In contrast, our recent study found lower perceived physical health, stress, coronavirus-related PTSD, fear of COVID-19, and depression, while higher in life satisfaction among unvaccinated that vaccinated adults [36], which is consistent with a fuzzy-trace theory [37]. Network analysis showed also that stress and anxiety levels have a main effect on depression and life satisfaction in both vaccinated and unvaccinated participants [36]. In this study, we will examine the relationship between fear of vaccination and mental-health dimensions, to check whether unvaccinated participants demonstrate better [36,37] or worse [34,35] mental-health. A systematic review and meta-analysis found moderate associations between fear of COVID-19 and mental health, including 0.42 for stress, 0.54 for anxiety, 0.40 for depression, and 0.24 for mental well-being [32]. Considering fear of COVID-19 vaccination as part of general fear of COVID-19, we will expect similar associations and hypothesize that fear of coronavirus vaccination will be related positively to fear of COVID-19, anxiety, depression, and stress, and negatively to life satisfaction and physical health.

## 2. Materials and Methods

### 2.1. Study Design

The prospective study was performed in a representative sample of 1723 young adults from Poland, Israel, Slovenia, and Germany, during two time-points of the third wave of the COVID-19 pandemic in 2021 (T1 = 19–26 February, and T2 = 26 May–9 June). The ARIADNA panel disseminated online surveys its registered individuals in Poland, Israel, Slovenia, and Germany, aged 20–40 (as an inclusion criterion). Four versions of the survey were prepared in each language (i.e., German, Hebrew, Polish, and Slovenian) from English, by a team of translators and mental-health experts, based on cross-cultural adaptation guidelines [38]. Participants voluntarily completed the survey in an average time of 21.52 min. (*SD* = 136.75). During T1, the data were collected from a sample of 2951 people, and during T2, from 1723 participants, giving a response rate of 58.42% in T2. This study is a part of the international project “Mental Health of Young Adults During the COVID-19 Pandemic in Poland, Germany, Slovenia, and Israel: A Longitudinal Study” [39].

### 2.2. Participants

The sample of 1723 young adults from Germany (*n* = 418, 24%), Israel (*n* = 428, 25%), Poland (*n* = 446, 26%), and Slovenia (*n* = 431, 25%), and aged between 20 and 40 years (*M* = 30.74, *SD* = 5.74) years participated in the two time-points of the study. In the total sample, 935 participants were women (54%), 1218 were single (71%), 420 were university or college students (24%), 1001 were child-free (58%), 1297 lived in towns or cities (75%), and 1218 were employed (71%).

### 2.3. Measurements

#### 2.3.1. Fear of Coronavirus Vaccination

The 7-item Fear of Coronavirus Vaccination Scale (FoCVVS) was adapted in the study from the Fear of COVID-19 Scale (FoCV-19S) developed by Ahorsu et al. [25], and the Hebrew [30] and Polish [40] language adaptations of FoCV-19S. The original FoCV-19S is a 7-item scale describing emotional and physiological symptoms of fear of coronavirus (e.g., “My heart races or palpitates when I think about getting coronavirus-19”). In FoCVVS, the word “coronavirus-19” was replaced with “vaccine for COVID-19” for all items. Participants rated on a 5-point Likert scale (from *Strongly disagree* = 1 to *Strongly agree* = 5) to what extent they agree with the symptom. The total score ranges from 7 to 35, with higher values indicating fear of COVID-19 vaccination. Internal reliability for the total score of FoCVVS was Cronbach’s α = 0.92 at T1 and Cronbach’s α = 0.93 at T2. 

#### 2.3.2. COVID-19 Vaccination Status

Vaccination status was assessed in the study using a single item: “Receiving the COVID-19 vaccine”, with response options *I am vaccinated* = 1, or *I am not vaccinated* = 0.

#### 2.3.3. Fear of COVID-19

The Fear of COVID-19 Scale (FoCV-19S) was developed by Ahorsu et al. [25] to assess the emotional and physiological response to the COVID-19 pandemic. The FoCV-19S is a 7-item scale with answers selected on a 5-point Likert scale (from *Strongly disagree* = 1 to *Strongly agree* = 5). The higher the scores (ranging from 7 to 35), the greater fear of COVID-19. In the present study, reliability assessed using Cronbach’s α coefficient was 0.91, 0.85, and 0.92 at T1 and 0.92, 0.87, and 0.93 at T2 for the total score, emotional, and physiological scales, respectively.

#### 2.3.4. Physical Health

Health-related quality of life was assessed using the General Self-Rated Health (GSRH), developed by DeSalvo et al. [41]. The GSRH includes two items from the standard general health survey (SF-12V). Participants rated on the 5-point Likert scale (from *Excellent* = 1 to *Poor* = 5) their health individually (item 1) and in comparison to others of the same age (item 2). Higher scores are interpreted as worse perceived health. Cronbach’s α was 0.90 and 0.89 during T1 and T2, respectively.

#### 2.3.5. Life Satisfaction

The 5-item Satisfaction With Life Scale (SWLS) was developed by Diener et al. [42] to assess a global cognitive aspect of subjective well-being. Participants rated on a 7-point Likert scale the extent of their agreement with a given statement (from *Strongly disagree* = 1 to *Strongly agree* = 7). The SWLS is a unidimensional instrument with scores ranging from 5 to 35 (the greater the score, the higher sense of satisfaction with life). The internal consistency of the SWLS in the current study was Cronbach’s α = 0.86 and 0.87 for T1 and T2, respectively. 

#### 2.3.6. Perceived Stress

The 10-item Perceived Stress Scale (PSS-10) is a unidimensional instrument to measure the frequency of stressful events in the last month [43], rated on a 5-point Likert scale (from *Never* = 0 to *Very often* = 4). The higher the scores (ranging from 0 to 40), the greater perceived stress occurred. In this study, Cronbach’s α was 0.83 during T1 and 0.83 at T2.

#### 2.3.7. Anxiety

The 7-item Generalized Anxiety Disorder (GAD-7) scale [44] assesses anxiety symptoms during the past two weeks, using a 4-point response scale (from *Not at all* = 0; to *Nearly every day* = 3). The GAD-7 is a unidimensional scale, with scores ranging between 0 and 21 (higher scores indicating a greater risk of general anxiety disorder). In this study, Cronbach’s α was 0.94 at T1 while 0.95 at T2.

#### 2.3.8. Depression

The 9-item Patient Health Questionnaire (PHQ-9) [45] was used to evaluate depressive symptoms in the last two weeks on a 4-point response scale (from *Not at all* = 0; to *Nearly every day* = 3). The PHQ-9 is a unidimensional toll with scores ranging from 0 to 27 (higher scores mean greater depression risk). This study’s reliability coefficients (Cronbach’s α) were 0.93 and 0.94 for T1 and T2, respectively.

#### 2.3.9. Sociodemographic Data

Sociodemographic data included questions regarding gender, age, place of residence (village, town, or city), student status (student or not a student), employment status (employed or unemployed), relationship status (coupled or single), and having children (having children or child-free).

### 2.4. Statistical Analysis

Descriptive statistics were calculated for the FoCVVS at T1 in each country-related sample separately, including a range of scores, mean (*M*), standard deviation (*SD*), median (*Mdn*), skewness, and kurtosis. Pearson’s correlations were used to examine associations between items of the FoCVVS at T1. Exploratory factor analysis (EFA), with a maximum likelihood (ML) estimation method, was performed 4 times, for samples from Germany (*n* = 418), Israel (*n* = 428), Poland (*n* = 446) and Slovenia (*n* = 431), during the first measurement (T1) using JASP software [46]. As a preliminary analysis, the Kaiser–Meyer–Oklin (KMO) measure and Bartlett’s Sphericity test were examined to explore whether the data’s properties are suitable for the EFA. The EFA was performed twice in each sample to test the one-factor (using Kaiser criterion) and alternative two-factor structure (using parallel factor analysis and oblique Promax rotation) of the fear of vaccination scale (FoCVVS). 

Confirmatory factor analysis (CFA) with maximum likelihood (ML) estimation method was performed for the total sample (*N* = 1723) during the second measurement (T2), using structural equation modeling (SEM) with AMOS for IBM SPSS software ver. 26 for Windows [47], with Master Validity plugins [48], to perform confirmatory composite analysis. The CFA was performed three times for the one-factor solution (Model 1), parallel two-factor solution (Model 2), and second-order two-factor solution (Model 3). Modification indices were used to improve model fit. Convergent validity [49,50] of each model was assessed based on the standardized factor loadings (β ≥ 0.70), Cronbach’s alpha reliability coefficient (α ≥ 0.70), composite reliability (CR ≥ 0.60), and average variance extracted (AVE ≥ 0.50). Discriminant validity was tested using the Fornell and Lacker [51] criterion and the heterotrait–monotrait (HTMT) ratio of correlation [52,53]. All structural models were evaluated using several goodness-of-fit criteria, including ML χ^2^, *df* and *p*-value (the ratio χ^2^/*df* < 2 is considered very good fit, between 2 and 3—good, and acceptable < 5), standardized root mean squared residual (SRMR ≤ 0.08 is acceptable), root mean square error of approximation (RMSEA; acceptable fit if ≤ 0.08, adequate fit if < 0.06, and good if 0.04), comparative fit index (CFI is acceptable if ≥ 0.90, and good if > 0.95) [54]. 

The measurement invariance (MI) was examined using multigroup CFA (MGCFA) to check whether FoCVVS scores in the latent variable and particular items varied across genders and countries. We conducted hierarchical tests for the invariance of measurement parameters, assuming more equality restrictions in each consecutive model in the following sequence: configural, metric, scalar, and strict MI models [55]. Configural invariance verified that the same CFA structure is valid in each gender or country group. Metric invariance assumes that factor loadings are equal across groups, examining whether participants across groups attribute the same meaning to the latent construct under study. The scalar invariance test of the null hypothesis examines whether the loadings and intercepts are constrained to be equal. Scalar invariance implies that the meaning of the construct (factor loadings) and the levels of the underlying items (intercepts) are equal in all groups. This means that scores on the latent variable are comparable across groups. Strict invariance constrained factor loadings, item intercept, and residual variances to be equal across groups. If strict invariance is confirmed, the latent construct is measured identically across groups [55,56]. Chen [55] suggests a change of CFI ≤ −0.005, supplemented by a change in RMSEA ≥ 0.010, as an indicator of non-invariance when the compared sample sizes are unequal.

Concurrent validity was examined using JASP software three times: (1) using a Student’s t-test for examining differences in fear of vaccination between vaccinated and unvaccinated people, separately for T1 and T2 (the effect size was assessed using Cohen’s d); (2) testing Pearson’s correlations between fear of vaccination and well-being dimensions in the total sample and among vaccinated and unvaccinated individuals; (3) performing multiple linear regression for vaccination fear as a dependent variable at T2, and all dimensions of well-being at T1 as potential predictor variables. Bootstrapping with the 1000 resampling method was used to increase the accuracy of measurement. 

## 3. Results

### 3.1. Exploratory Factor Analysis for Fear of Vaccination Scale during T1

Descriptive statistics for seven items of the FoCVVS, including range, mean, standard deviation, skewness, kurtosis, and Pearson’s correlations, are presented in Tables S1–S4 (in the Supplementary Materials) for the samples from Germany (*n* = 418), Israel (*n* = 428), Poland (*n* = 446), and Slovenia (*n* = 431), respectively. The descriptive statistics showed an appropriate distribution of the data for parametric tests. Significant and positive correlations (above 0.30) between seven items of the FoCVVS were demonstrated in all four countries, ranging between 0.51 and 0.86 for Germany, 0.50 and 0.83 for Israel, 0.47 and 0.87 for Poland, and 0.36 and 0.82 for Slovenia. 

The EFA was replicated in each country (e.g., Germany, Israel, Poland, and Slovenia) to explore the factor structure of the FoCVVS dependent on language. The results are presented in Table 1. KMO was 0.884, 0.884, 0.874, and 0.866 for samples from Germany, Israel, Poland, and Slovenia, respectively. Bartlett’s Sphericity test was significant at *p* < 0.001 for all samples, and equaled χ^2^(21) = 2197.43, χ^2^(21) = 2418.24, χ^2^(21) = 2463.82, and χ^2^(21) = 2019.31, for samples from Germany, Israel, Poland, and Slovenia, respectively. All these tests showed that factor analysis is appropriate to perform. 

**Figure 1:**
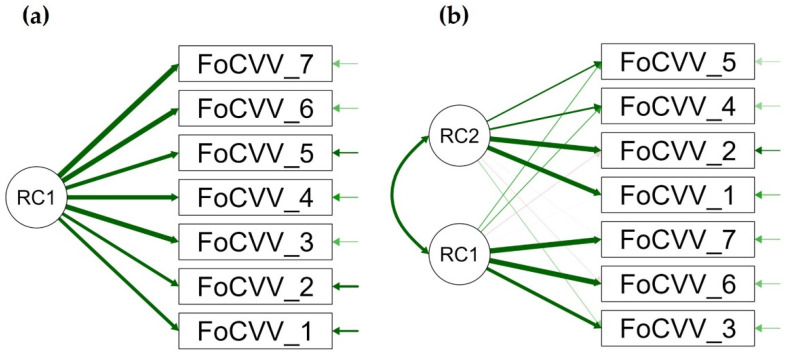
Path diagram for (**a**) Model 1 with a one-factor solution and (**b**) Model 2 with a two-factor solution of the fear of vaccination scale. FoCVV = item of the Fear of Coronavirus Vaccination, RC = rotated component.

One-factor solution (Model 1) was found using the Kaiser criterium (eigenvalues above 1) in all four samples, with factor loading ranging 0.59–0.89, 0.69–0.87, 0.70–0.86, and 0.60–0.88, in the German, Israeli, Polish, and Slovenian samples, respectively (see Table 1, and Figure 1a for more details). Uniqueness ranged 0.21–0.65, 0.24–0.53, 0.26–0.50, and 0.22–0.63, for people from Germany, Israel, Poland, and Slovenia, respectively (Table 1). Proportion of variance explained was 0.63% in German (eigenvalue 4.38), 0.64% in Israeli (eigenvalue 4.45), 0.62% in Polish (eigenvalue 4.37), and 0.57% in Slovenian (eigenvalue 4.00) samples. In German sample, one-factor solution showed poor fit, RMSEA = 0.230 (0.205–0.248), TLI = 0.79, BIC = 227.88, χ^2^(14) = 312.37, *p* < 0.001. In Israeli sample, RMSEA was 0.280 (0.258–0.301), TLI = 0.71, BIC = 395.12, χ^2^(14) = 479.95, *p* < 0.001. In Polish sample, RMSEA was 0.290 (0.268–0.310), TLI = 0.68, BIC = 449.33, χ^2^(14) = 534.73, *p* < 0.001. In Slovenian sample, RMSEA was 0.250 (0.233–0.276), TLI = 0.71, BIC = 319.11, χ^2^(14) = 404.03, *p* < 0.001. Model 1 fit statistics were overall not acceptable in all four language samples.

Parallel exploratory factor analysis with the oblique Promax rotation method was used to determine an appropriate number of factors in the next step. The analysis showed a two-factor solution in all four countries (see Model 2 in Table 1, and Figure 1b). Factor 1 comprised items 3, 5, 6, and 7 in Germany (eigenvalue 4.48), items 3, 6, and 7 in Israel (eigenvalue 4.57), items 1, 2, 4, and 5 in Poland (eigenvalue 4.49), and items 3, 6, and 7, in Slovenia (eigenvalue 4.13). Factor 2 included items 1, 2, and 4 in Germany (eigenvalue 0.64), 1, 2, 4, and 5 in Israel (eigenvalue 0.75), items 3, 6, and 7 in Poland (eigenvalue 0.74), and items 1, 2, 4, and 5 in Slovenia (eigenvalue 0.78). Apart from the sample from Germany (item 5 was loaded differently), the two-factor structure from the total sample was replicated in three other countries, namely Israel, Poland, and Slovenia. Uniqueness was better in Model 2 for all four samples, and more variance was explained by Model 2 in Germany (F1 = 0.64%, F2 = 0.09%), Israel (F1 = 0.65%, F2 = 0.11%), Poland (F1 = 0.64%, F2 = 0.11%), and Slovenia (F1 = 0.59%, F2 = 0.11%). Model fit statistics were better in Model 2 (compared to Model 1) for the sample from Germany [RMSEA = 0.080 (0.046–0.108), TLI = 0.98, BIC = −21.11, χ^2^(8) = 27.18, *p* < 0.001], Israel [RMSEA = 0.070 (0.040–0.103), TLI = 0.98, BIC = −23.57, χ^2^(14) = 24.90, *p* < 0.01], Poland [RMSEA = 0.100 (0.068–0.126), TLI = 0.96, BIC = −8.16, χ^2^(8) = 40.64, *p* < 0.001], and Slovenia [RMSEA = 0.080 (0.052–0.112), TLI = 0.97, BIC = −18.13, χ^2^(8) = 30.40, *p* < 0.001].

### 3.2. Confirmatory Factor Analysis for Fear of Vaccination Scale during T2

Table 2 shows the results of CFA for Model 1, Model 2, and Model 3. All scores demonstrate adequate validity for all three models, regarding a high value of standardized factor loadings, good internal consistency assessed by the Cronbach’s alpha reliability coefficient, well indicator reliability measured by composite reliability, and adequate convergent validity achieved by average variance extracted [49,50]. 

Discriminant validity was tested using the Fornell and Lacker [51] criterion, comparing the square root of the AVE with the correlation of latent constructs. The square root of AVE for Factor 1 was 0.84, while for Factor 2 was 0.893, which was a greater value than the correlations between Factor 1 and Factor 2 (*r* = 0.80, *p* < 0.001), confirming good discriminant validity for two-factor solution [53]. Divergent validity was also adequately using the heterotrait–monotrait (HTMT) ratio of correlation [52], which was 0.80 in the study (so lower than the threshold of 0.85, as recommended by Kline [53]. The two factors of the FoCVVS are distinct constructs from each other.

**Figure 2:**
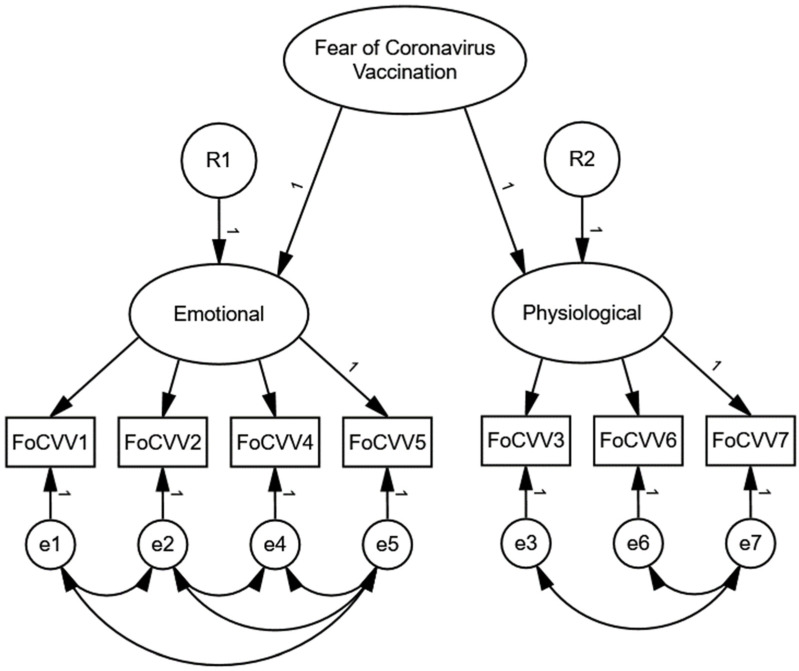
Model 3 plot. FoCVV = item of the Fear of Vaccination, R = error for a latent variable, e = error for an observed variable.

The following fit indices were examined in the study to test the construct validity of models: χ^2^/*df* (relative chi-square), comparative fit index (CFI), standardized root mean squared residual (SRMR), and root mean square error of approximation (RMSEA). The fit indices were poor in Model 1, while they showed an adequate structure in Model 2 and Model 3 (see Table 3), considering combinational rules with RMSEA < 0.06, SRMR < 0.08, and CFI > 0.95 [54]. Furthermore, the Akaike information criterion (AIC) and the Bayesian information criterion (BIC) were used to compare models. Model 1 differed strongly from Models 2 and 3, showing much poorer Model 1 complexity than Model 2 and Model 3. Weak evidence was found for better performance of Model 3 in comparison to Model 2 (AIC and BIC were slightly lower in Model 3). Therefore, the second-order two-factor Model 3 was selected for further multigroup analysis since the best fitted for the data (see Figure 2 for more details).

As a sensitivity analysis, we conducted CFA separately for each country-related sample at the second measurement (T2). The EFA showed at T1 that item 5 (“When I watch news and stories about a vaccine for COVID-19 on social media, I become nervous or anxious”) loaded to physiological symptoms of fear of coronavirus vaccination in the German sample, in contrast to other languages, in which item 5 was loaded in emotional symptoms. Therefore, in the German sample, the CFA was conducted twice to compare Model 3 presented above (items 1, 2, 4 and 5 in the emotional component, and items 3, 6 and 7 in physiological) with Model 2 found in the German sample in the EFA at T1 (items 1, 2 and 4 in the emotional component, and items 3, 5, 6 and 7 in the physiological component). As shown in Table 4, Model 3 in CFA presented better fit indices than Model 2 in EFA for the German language. Additionally, Model 3 in CFA showed a good fit for the other languages.

### 3.3. Multigroup Analysis of Invariance

The measurement invariance across genders and languages was examined in the FoCVVS scores during T2 using multigroup CFA (MGCFA). Results are shown in Table 5. The baseline model was developed separately for the total sample (Model 0, Table 5) and for each gender (Model A = Women, B = Men) and language group (Model C = German, D = Hebrew, E = Polish, F = Slovenian). The baseline model for the total sample (Model 0), separate models for women (Model A) and men (Model B), and configural Model 1 for MI across gender, were acceptable regarding SRMR, RMSEA, CFI, and TLI, but insufficient, when using the χ^2^ test (*p* < 0.05). However, the fit indices were slightly better for women than for men. Configural measurement invariance was confirmed, which means that the pattern of the factor loadings on the latent variables is similar across genders. The MI metric Model 2 (Table 5) was unchanged considering SRMR while improved regarding χ^2^/*df*, RMSEA, CFI, and TLI. Since fit indices were not worse in the metric Model 2 compared to the configural Model 1, metric invariance was supported in the study, indicating that constraining the loadings across groups does not significantly affect the model fit. Scalar invariance was examined by comparing Model 3 with Model 2 (Table 5). The item intercepts (means) are similar across genders since no worsening in fit indices was found in the study. Scalar invariance across genders was confirmed. The strict invariance was examined by comparing Model 4 with Model 3 (Table 5). Some fit indices (χ^2^/*df* and RMSEA) slightly improved, some worsened lightly (SRMR and TLI), whereas CFI was unchanged. Since significant differences were not found between Model 4 and Model 3, strict invariance between women and men was also supported, which means that there is an equivalence of item residuals of metric and scalar invariant items of the FoCVVS.

A multiple group analysis without equality constraints was also performed for each language (Model C, D, E, and F in Table 5). Baseline models showed a very good fit for German (Model C) and Hebrew (Model D), while an acceptable fit for Polish (Model E) and Slovenian (Model F, Table 5). The configural MI Model 5 (Table 5) perfectly fits all stats, including χ^2^/*df*, SRMR, RMSEA, CFI, and TLI. The configural MI Model 5 differed significantly from the metric MI Model 6 (Table 5), which means that factor loadings were unequal in four languages. The metric MI Model 6 differed significantly from scalar MI Model 7 (Table 5). Furthermore, a significant difference was found between scalar MI Model 7 and strict MI Model 8 (Table 5), indicating that the intercepts and residual variance differed across languages. The hypothesis about metric, scalar, and strict measurement invariance across languages was not supported in the study.

Furthermore, one-way analysis of variance (ANOVA) showed that language samples differ in mean scores, indicating that FoCVV was usually lower in samples from Israel than from Slovenia, Germany, and Poland (Table S5, Supplementary Materials). The CFA Model 3 was replicated for four languages separately: German, Hebrew, Polish, and Slovenian. A comparison of factor loadings in Model 3 is shown in Table S6 in Supplementary Materials. Overall, the analysis showed that the strongest loadings are presented in Polish, German and Slovenian, and the weakest in the Israeli samples.

### 3.4. Concurrent Validity of the FoCVVS

Concurrent validity was examined by comparing vaccinated and unvaccinated participants in all dimensions of fear of vaccination and well-being, separately at T1 and T2. Considering T1 (Table S7, Supplementary Materials), unvaccinated people scored significantly higher than those vaccinated in fear of vaccination (general, emotional, and physiological), perceived stress, and anxiety symptoms, and they scored lower in fear of COVID-19 and physical health (which means better health). However, when T2 was analyzed (Table S8, Supplementary Materials), unvaccinated people showed significantly higher fear of vaccination and better physical health (lower scores) than vaccinated people. Still, none of the dimensions of well-being differentiated these groups. 

Pearson’s correlations were performed to examine longitudinal associations between fear of vaccination T2 and well-being dimensions T1 (Table S9, Supplementary Materials). In the total sample (*N* = 1723), fear of general vaccination scores at T2 was related positively and moderately to all three scales of vaccination fear and weakly to fear of COVID-19 three scales, stress, anxiety, and depression. Similar associations were found for physiological and emotional subscales, except for a negative association between emotional symptoms of fear of vaccination and physical health (higher fear was presented in those with better health). The same pattern of relationships was demonstrated among vaccinated and unvaccinated participants in the total sample. Still, the association of the FoCVVS total score with physical health was positive in the vaccinated sample while negative in the unvaccinated group. We can conclude that vaccination status moderates the association between fear of vaccination and physical health in the total sample. Similar associations with the general scale of fear of vaccination were shown for both emotional and physiological subscales, including the pattern of a relationship with physical health. Life satisfaction was unrelated to fear of vaccination, except for a marginal negative association with the emotional subscale. 

Finally, multiple linear regression analysis was performed to examine the predictors of fear of vaccination during T2 longitudinally among well-being variables at T1 (Table 6). The results showed that fear of vaccination at the first time-points, fear of COVID-19, and vaccination status were significant predictors of fear of vaccination at T2. Higher scores of fear of vaccination were related to higher fear of COVID-19 and the unvaccinated status of participants (negative association). None of the other physical or mental-health dimensions were related to the fear of vaccination. Additionally, the interaction between physical health and vaccination status was not significant. The model explained 49% fear of vaccination, *R* = 0.70, *R*^2^ = 0.49, *F*(8,1283) = 153, *p* < 0.001.

## 4. Discussion

This study confirmed that the fear of vaccination scale has good psychometric properties, and this is a valid measure to examine emotional and physiological symptoms of fear of vaccination in German, Hebrew, Polish, and Slovenian languages. The two-factor structure (with items 1, 2, 4, and 5 on the emotional symptoms scale and items 3, 6, and 7 on the physiological symptoms) was replicated and confirmed in Hebrew, Polish, and Slovenian languages using EFA at T1 measurement and CFA at T2. The two-factor structure with emotional and physiological components is consistent with previous studies regarding the original Fear of COVID-19 Scale [29,30,31]. However, this structure was not confirmed in the German language using EFA at T1 since item 5 loaded into physiological symptoms of the FoCVVS, instead of emotional symptoms as in the other languages and previous studies [29,30,31]. Previously, Malas and Tolsá [24] excluded item 5 from the newly adapted FoCV-19S to Vaccination Fear Scale (VFS-6) because of unacceptable loading weight (<0.50). It is important to note that in the Spanish language, item 5 was almost equally loaded to factors F1 and F2 (0.46 and 0.44, respectively). Therefore, more research is necessary to examine the FoCVVS in various populations to test whether item 5 should be removed from the scale in the future, avoiding its unclear role in the structure. On the other hand, CFA at T2 showed a better fit for Model 3, shared for all other countries in this study, than for Model 2 found in EFA during T1. Therefore, we can conclude that a two-factor solution with original emotional and physiological symptoms scales showed the best structure for the FoCVVS.

Comparing one-factor and two-factor models using EFA in four language-related samples, the acceptable fit was shown only for the two-factor solution. However, when CFA was conducted, the hierarchical second-order Model 3 was better than one-factor Model 1 and parallel two-factors Model 2. Therefore, three composite scores can be used in future studies: (1) a general scale of the FoCVVS (items 1–7); (2) an emotional symptoms subscale (items: 1, 2, 4, and 5), and (3) physiological symptoms subscale (items: 3, 6, and 7). Fit indices were appropriate for the second-order two-factor structure, and both convergent and discriminant validity of the general factor and both subscales were very good. Furthermore, gender invariance was confirmed in the study, indicating that women and men equally understand the content of the FoCVVS. 

In contrast, country heterogeneity was found in metric, scalar, and strict measurement invariance analyses, showing that the scale is sensitive to cross-cultural contexts. The multigroup CFA at T2 showed configural measurement invariance for the expected Model 3 structure (with items 1, 2, 4, and 5 on the emotional symptoms scale, and items 3, 6, and 7 on the physiological symptoms scale) across countries. The same conclusion was derived from replicated CFA Model 3 separately for each language-related sample since the CFA showed good fit indices for German, Hebrew, Polish, and Slovenian languages. Therefore, we can state that the two-factor structure is the same, independent of country and gender. However, further metric, scalar, and strict invariance analyses showed that Model 3 differs across languages. We found the strongest loadings in Polish, German, and Slovenian languages and the weakest in Hebrew. Regarding intercepts of particular FoCVV items, the mean score of fear of vaccination items was usually significantly lower in samples from Israel than from Slovenia, Germany, and Poland. A systematic review and meta-analysis showed previously that mean scores of the total fear of COVID-19 (measured using FoCV-19S) differs across continents, with the highest levels in Asia and the lowest in Australia [33]. The differences in fear of COVID-19 mean scores are related to restriction levels in different countries and various methods of coping with the pandemic. The present results may also be related to the number of vaccinated people [12]. Vaccination frequency was more than twice as high in Israel as in Germany, Poland, and Slovenia during the data collection, which to some extent may depend on the quality and degree of organization of health services in different countries, greater or lesser trust to the health service, public administration, and institutions, or differences in the dissemination of knowledge about vaccinations and promoting pro-health behavior. Our previous research showed that trust in institutions was a positive predictor of FoCV-19S, and this relationship was moderated by country [57]. More research is required to examine associations between fear of vaccination and country-specific health-related variables worldwide to explain cross-cultural differences.

Previous research showed that countries and geographic regions of the world differ in vaccination prevalence rates [10,11]. Pandey et al. [58] showed that vaccination is related to many factors that affect populations’ physical and mental health worldwide. Emotional and behavioral reactions of individuals and communities to the COVID-19 pandemic were determined by stress related to changes in work and school places and numerous restrictions during following lockdowns (like social distancing). Several social determinants and disparities contribute to differences in mental stress of various populations, including the disproportionate burden of COVID-19 on racial and ethnic minorities and undocumented immigrants with access to quality health care, safe housing, education, or social and community context. Disproportions of disadvantaged groups in various countries may affect multiple emotional and physiological responses to COVID-19 vaccination, including increased fear of vaccination. Beside socio-economic factors, psychological factors also contribute to vaccine hesitancy, such as health-related and protective behavior, beliefs, perception, and attitude toward vaccination. It is plausible that all these factors are also country-specific. Unfortunately, international studies are sparse and lack reviews and meta-analyses in the global context. Further cross-cultural research should collect various socio-economic, political, and psychological variables to explain vaccination, especially in the context of fear of vaccination.

The concurrent validity of the FoCVVS showed that the scale differs between vaccinated and unvaccinated participants and is associated with several dimensions of well-being. This study showed that unvaccinated participants scored higher in the total fear of vaccination and subscales of emotional and physiological symptoms of fear than those vaccinated (at both time-points T1 and T2). In addition, vaccination status was a significant predictor of fear of vaccination in the regression analysis. These results are consistent with previous studies, showing that fear of vaccination-related harm and side effects is the crucial reason to refuse vaccination [4,6,7,8,9,13,14,15,16,17,18,19]. Alzubaidi et al. [59] recommended reducing fears about the adverse effects of vaccination as a prevention or intervention strategy to increase COVID-19 vaccination uptake. Future promotion actions in mass media should present reliable statistics and data on the prevalence of side effects and be more focused on the beneficial effects of vaccination to decrease the fear of vaccination in populations. 

This study indicates that during the first time-point, unvaccinated people had better physical health and fewer emotional symptoms of fear of COVID-19. Still, simultaneously they had higher stress and anxiety compared to vaccinated participants. These results are partially consistent with other evidence. Higher stress and anxiety were reported in unvaccinated than in vaccinated people previously [34,35]. Considering the association between fear of vaccination and fear of COVID-19, both current and previous research are inconclusive. Babicki et al. [34] found higher fear of COVID-19 in unvaccinated, and Galanis et al. [14] in vaccinated individuals, which seems more consistent with our study. Furthermore, Lo Moro et al. [60] did not find a significant relationship between fear of COVID-19 and hesitancy to receive vaccination against COVID-19. On the other hand, correlation analysis showed in this study a positive association between fear of vaccination T2 and fear of COVID-19 T1 in both vaccinated and unvaccinated people. Finally, fear of COVID-19 T1 was a positive predictor of fear of vaccination T2, supporting here longitudinally the positive association between these variables. It seems rational that negative emotions such as stress, anxiety, and fear are generalized into various health-related dimensions, such as vaccination or coronavirus risk. However, we need more research to check whether the inconsistency between studies is country-specific, related to various phases of the pandemic, or other factors. 

Better physical health among unvaccinated adults than vaccinated was found at T1 and T2. In contradiction to our findings, previous studies have shown better health among vaccinated people than in unvaccinated [34,35]. Our result may mean that unvaccinated individuals are generally physically healthy and may think not to change this status quo, which is consistent with the fuzzy-trace theory [37]. Indeed, our previous research showed that vaccinated people demonstrate higher exposure to COVID-19, stress, coronavirus-related PTSD, fear of COVID-19, and depression, and worse physical health and life satisfaction, compared to unvaccinated individuals [36]. Vaccination increases anxiety and stress in unvaccinated people, threatening their “feel okay” status. Therefore, they have a negative attitude to vaccination and tend to avoid thinking about vaccination. Some conspiracy theories and other irrational explanations may help decrease cognitive discrepancy and sustain the decision not to vaccinate. Therefore, conspiracy theories and myths on vaccination should be systematically debunked by showing scientific evidence in mass media to promote vaccination. Additionally, media and schools should explain mechanisms and learn the best strategies on how to cope with misinformation.

The FoCVVS can be used to measure the emotional and physiological symptoms of fear of vaccination. People who recognize symptoms of fear can be treated to mitigate or eliminate this state by such strategies as increasing sound scientific knowledge about vaccination, training of positive thinking about the beneficial effects of vaccination, relaxation linked with visualization of vaccination in a safe setting, learning of breath techniques, looking for support and talking about experiences of vaccination with healthcare experts, friends or/and family members, or looking for the support group. All these strategies can help to decide on vaccination among undecided and hesitating people.

Although we showed strong evidence that fear of vaccination can be effectively measured using FoCVVS, some limitations of this study must also be described. First, the research can be generalized to young adults between 20 and 40. It is not certain whether the FoCVVS will also be suitable for younger or older populations. Additionally, the study was primarily performed in European countries, so more international studies are necessary to replicate the validity of this tool in various geographic regions of the world. The study was conducted online, which may be related to some bias. Future studies should consider multiple study methods (e.g., paper-and-pencil, telephone-based interviews) to triangulate the present results. We used vaccination status and several dimensions of well-being to examine concurrent validation of the FoCVVS. Future studies may test associations of the FoCVVS with motives, perceptions, beliefs, attitudes, and other health-related or preventive behaviors.

## 5. Conclusions

The FoCVVS is a reliable and validated instrument to measure emotional and physiological responses to vaccination, which was confirmed in four languages: German, Hebrew, Polish, and Slovenian (Appendix A, Table A1, Table A2, Table A3, Table A4 and Table A5). The FoCVVS is recommended to be widely used worldwide for verifying the validity of this scale in various cross-cultural and social contexts and to examine the fear of COVID-19 as one of the crucial factors determining vaccination. Based on the FoCVVS measurement, prevention and intervention programs focused on alleviating the fear of vaccination should be implemented at universities, workplaces, and healthcare services. Unvaccinated people demonstrate higher fear of vaccination than vaccinated individuals, so vaccination promotion programs should be focused on decreasing the fear of vaccination and showing the beneficial effects of vaccination. 

## Figures and Tables

**Table 1:** Factor loadings for Model 1 (one-factor solution) and Model 2 (two-factor solution) of the Fear of Coronavirus Vaccination Scale for samples from Germany, Israel, Poland, and Slovenia.

Country	Item	Model 1		Model 2		
		1 Factor	Uniq.	Factor 1	Factor 2	Uniq.
Germany	FoCVV_1	0.68	0.54		0.92	0.20
	FoCVV_2	0.59	0.65		0.85	0.37
	FoCVV_3	0.83	0.31	0.71		0.33
	FoCVV_4	0.78	0.39		0.48	0.35
	FoCVV_5	0.82	0.33	0.57		0.35
	FoCVV_6	0.87	0.23	0.99		0.16
	FoCVV_7	0.89	0.21	1.01		0.13
Israel	FoCVV_1	0.69	0.53		0.94	0.20
	FoCVV_2	0.70	0.51		0.99	0.14
	FoCVV_3	0.83	0.30	0.87		0.26
	FoCVV_4	0.79	0.37		0.43	0.37
	FoCVV_5	0.77	0.41		0.50	0.38
	FoCVV_6	0.87	0.24	0.93		0.18
	FoCVV_7	0.87	0.24	0.95		0.16
Poland	FoCVV_1	0.71	0.50	0.88		0.26
	FoCVV_2	0.70	0.50	0.92		0.23
	FoCVV_3	0.84	0.29		0.66	0.30
	FoCVV_4	0.78	0.39	0.65		0.33
	FoCVV_5	0.75	0.44	0.64		0.39
	FoCVV_6	0.85	0.28		0.94	0.14
	FoCVV_7	0.86	0.26		0.95	0.11
Slovenia	FoCVV_1	0.65	0.58		0.80	0.30
	FoCVV_2	0.60	0.63		0.93	0.22
	FoCVV_3	0.87	0.24	0.74		0.27
	FoCVV_4	0.74	0.46		0.57	0.38
	FoCVV_5	0.63	0.60		0.42	0.57
	FoCVV_6	0.82	0.33	0.93		0.22
	FoCVV_7	0.88	0.22	0.92		0.14

Note. FoCVV_1 = number of item of the Fear of Coronavirus Vaccination Scale.; Uniq. = uniqueness.

**Table 2:** Results of the confirmatory factor analysis for Fear of Coronavirus Vaccination Scale.

Model	Latent Construct	Item	Loadings	α	CR	AVE
1	Fear of Vaccination	FoCVV_1	0.72 ***	0.93	0.93	0.65
		FoCVV_2	0.71 ***			
		FoCVV_3	0.86 ***			
		FoCVV_4	0.81 ***			
		FoCVV_5	0.79 ***			
		FoCVV_6	0.86 ***			
		FoCVV_7	0.89 ***			
2	Factor 1 Physiological	FoCVV_3	0.90 ***	0.93	0.91	0.71
		FoCVV_6	0.86 ***			
		FoCVV_7	0.91 ***			
	Factor 2 Emotional	FoCVV_1	0.78 ***	0.90	0.92	0.80
		FoCVV_2	0.77 ***			
		FoCVV_4	0.91 ***			
		FoCVV_5	0.91 ***			
3	Fear of Vaccination	Factor 1	0.86 ***	0.84	0.89	0.80
		Factor 2	0.93 ***			
	Factor 1 Physiological	FoCVV_3	0.90 ***	0.93	0.91	0.71
		FoCVV_6	0.86 ***			
		FoCVV_7	0.91 ***			
	Factor 2 Emotional	FoCVV_1	0.78 ***	0.90	0.92	0.80
		FoCVV_2	0.77 ***			
		FoCVV_4	0.91 ***			
		FoCVV_5	0.91 ***			

Note. α = Cronbach’s alpha, CR = composite reliability, AVE = average variance extracted, FoCVV = item of the Fear of Coronavirus Vaccination. *** *p* < 0.001.

**Table 3:** Model fit indices.

Model	χ^2^	*df*	χ^2^/*df*	CFI	SRMR	RMSEA	AIC	BIC
1 (one-factor)	1543.504	14	110.250	0.847	0.090	0.252	1571.504	1647.829
2 (two-factor)	32.784	6	5.464	0.997	0.011	0.051	76.784	196.724
3 (second-order two-factor)	32.784	6	5.464	0.980	0.011	0.051	76.058	195.998

Note. CFI = Comparative Fit Index, SRMR = standardized root mean squared residual, RMSEA = root mean squared error of approximation, AIC = Akaike information criterion, BIC = Bayesian information criterion.

**Table 4:** Comparison of model fit indices between four languages.

Model Fit Index	German		Hebrew Model 3 CFA	Polish Model 3 CFA	Slovenian Model 3 CFA
	Model 3 CFA	Model 2 EFA			
χ^2^ (*df*)	6.14 (5)	33.53 (7)	12.21(6)	23.80 (6)	17.00 (5)
χ^2^ *p*-value	0.29	<0.001	0.06	<0.001	0.01
CFI	1.00	0.99	1.00	0.99	0.99
TLI	1.00	0.97	0.99	0.98	0.97
RMSEA	0.02	0.10	0.05	0.08	0.07
RMSEA 90% CI LB	<0.001	0.06	<0.001	0.05	0.04
RMSEA 90% CI UB	0.08	0.13	0.09	0.12	0.12
RMSEA *p*-value	0.75	0.01	0.46	0.06	0.12
SRMR	0.01	0.02	0.01	0.01	0.02
AIC	7151.40	7174.80	6402.36	7181.57	7732.84
BIC	7244.22	7259.54	6491.66	7271.78	7826.36

Note. CFI = Comparative Fit Index; TLI = Tucker-Lewis Index; RMSEA = root mean square error of approximation; CI = confidence interval; LB = lower bound; UB = upper bound; SRMR = standardized root mean square residual; AIC = Akaike Information Criterion; BIC = Bayesian Information Criterion, CFA = confirmatory factor analysis, EFA = exploratory factor analysis.

**Table 5:** Results of the multigroup confirmatory factor analysis to examine measurement invariance across genders and languages.

Models		χ^2^ (*df*)	χ^2^/df	*p*	SRMR	RMSEA (90% CI)	CFI
0	Baseline	32.058 (6)	5.343	<0.001	0.009	0.050 (0.034–0.068)	0.980
Gender invariance							
A	Women	18.952 (6)	3.159	<0.01	0.009	0.048 (0.025–0.073)	0.982
B	Men	26.886 (6)	4.481	<0.001	0.013	0.067 (0.042–0.093)	0.965
1	MI configural	47.339 (12)	3.945	<0.001	0.009	0.041 (0.029–0.054)	0.996
2	MI metric	50.790 (17)	2.988	<0.001	0.009	0.034 (0.023–0.045)	0.997
3	MI scalar	50.791 (18)	2.822	<0.001	0.009	0.033 (0.022–0.043)	0.997
4	MI strict	54.341 (20)	2.717	<0.001	0.010	0.032 (0.022–0.042)	0.997
Country invariance							
C	German	6.042 (6)	1.007	0.419	0.006	0.004 (0.000–0.064)	1.000
D	Hebrew	9.743 (6)	1.624	0.136	0.010	0.038 (0.000–0.080)	0.989
E	Polish	21.968 (6)	3.661	0.001	0.016	0.077 (0.044–0.113)	0.956
F	Slovenian	16.387 (6)	2.731	0.012	0.017	0.063 (0.028–0.101)	0.966
5	MI configural	56.811(24)	2.367	<0.001	0.014	0.028 (0.019–0.038)	0.997
6	MI metric	159.573 (39)	4.092	<0.001	0.019	0.042 (0.036–0.049)	0.988
7	MI scalar	436.782 (60)	7.280	<0.001	0.019	0.060 (0.055–0.066)	0.962
8	MI strict	499.097 (69)	7.233	<0.001	0.022	0.060 (0.055–0.065)	0.957

Note. SRMR = standardized root mean square residual; RMSEA = root mean square error of approximation; CI = confidence interval; CFI = Comparative Fit Index; MI = measurement invariance.

**Table 6:** Multiple linear regression for fear of coronavirus vaccination (*N* = 1723).

			Bootstrap 95% CI					Bootstrap 95% CI	
Predictor	*b*	*SE b*	Lower	Upper	*t*	*p*	β	Lower	Upper
Intercept	5.38	1.11	3.20	7.56	4.84	<0.001			
Fear of coronavirus vaccination	0.57	0.03	0.52	0.62	22.10	<0.001	0.56	0.51	0.61
Fear of COVID-19	−0.02	0.08	−0.19	0.14	−0.27	0.788	−0.01	−0.05	0.04
Physical health	0.09	0.03	0.03	0.15	3.05	0.002	0.08	0.03	0.13
Life satisfaction	0.01	0.03	−0.05	0.06	0.30	0.761	0.01	−0.04	0.05
Stress	0.02	0.03	−0.04	0.09	0.70	0.485	0.02	−0.04	0.08
Anxiety	−0.02	0.06	−0.14	0.10	−0.26	0.797	−0.01	−0.10	0.08
Depression	0.04	0.05	−0.06	0.13	0.78	0.436	0.03	−0.05	0.12
Vaccination status	−2.91	0.32	−3.54	−2.29	−9.15	<0 .001	−0.40	−0.48	−0.31

Note. CI = confidence interval.

## Data Availability

This study is a part of an international research project, “Mental health of young adults during the COVID-19 pandemic in Poland, Germany, Slovenia, and Israel: A Longitudinal Study” [39], registered at the Center for Open Science (OSF). The datasets used and analyzed during the current study are available from the corresponding author upon reasonable request.

## Supplementary Materials

The following supporting information can be downloaded at: https://www.mdpi.com/article/10.3390/ijerph191811587/s1, Table S1: Descriptive statistics for the sample of adults from Germany (*n* = 418); Table S2: Descriptive statistics for the sample of adults from Israel (*n* = 428); Table S3: Descriptive statistics for the sample of adults from Poland (*n* = 446); Table S4: Descriptive statistics for the sample of adults from Slovenia (*n* = 431); Table S5: One-way ANOVA results for the Fear of Coronavirus Vaccination Scale across four languages; Table S6: The regression loadings in Model 3 across four languages; Table S7: Differences in fear of coronavirus vaccination and well-being dimensions between unvaccinated and vaccinated participants during T1 of the third wave of the COVID-19 pandemic; Table S8: Differences in fear of vaccination and well-being dimensions between unvaccinated and vaccinated participants during the T2 of the third wave of the COVID-19 pandemic; Table S9: Pearson’s correlations between three scales of fear of vaccination (general, emotional, and physiological) during T2 and well-being dimensions at T1, stratified into the total sample of vaccinated and unvaccinated participants.

## Appendix A

***SCORING*:** A total score is calculated by adding up each item score (items 1–7); Emotional symptoms of fear subscale (items 1, 2, 4, and 5); Physiological symptoms of fear subscale (items 3, 6, and 7).

**Table A1:** Fear of Coronavirus Vaccination Scale (FoCVVS) in the English language. ***Attitude towards the Vaccination against COVID-19.*** Mark your answers using 1–5: 1 = completely disagree; 2 = disagree; 3 = neither agree nor disagree; 4 = agree; 5 = completely agree.

ITEM	CONTENT
FoCVV_1	I am most afraid of a vaccine for COVID-19.
FoCVV_2	It makes me uncomfortable to think about a vaccine for COVID-19.
FoCVV_3	My hands become clammy when I think about a vaccine for COVID-19.
FoCVV_4	I am afraid of losing my life because of a vaccine for COVID-19.
FoCVV_5	When I watch news and stories about a vaccine for COVID-19 on social media, I become nervous or anxious.
FoCVV_6	I cannot sleep because I’m worried about taking a vaccine for COVID-19.
FoCVV_7	My heart races or palpitates when I think about taking a vaccine for COVID-19.

**Table A2:** Fear of Coronavirus Vaccination Scale (FoCVVS) in the German language. ***Einstellung zum COVID-19-Impfstoff.*** Markieren Sie Ihre Antworten auf der Skala von 1 bis 5: 1 = Ich stimme überhaupt nicht zu; 2 = Ich stimme nicht zu; 3 = Neutral; 4= Ich stimme zu; 5 = Ich stimme voll und ganz zu.

FoCVV_1	Ich habe größte Angst vor einem Impfstoff gegen COVID-19.
FoCVV_2	Ich fühle mich unbehaglich, wenn ich über einen Impfstoff gegen COVID-19 nachdenke.
FoCVV_3	Meine Hände werden feucht, wenn ich über einen Impfstoff gegen COVID-19 denke.
FoCVV_4	Ich habe Angst, mein Leben wegen eines Impfstoffs gegen COVID-19 zu verlieren.
FoCVV_5	Wenn ich Nachrichten und Geschichten über einen Impfstoff gegen COVID-19 in den sozialen Medien sehe, werde ich nervös oder ängstlich.
FoCVV_6	Ich kann nicht schlafen, weil ich mir Sorgen mache, einen Impfstoff gegen COVID-19 nehmen zu müssen.
FoCVV_7	Mein Herz rast und klopft, wenn ich daran denke, einen Impfstoff gegen COVID-19 nehmen zu müssen.

**Table A3:** Fear of Coronavirus Vaccination Scale (FoCVVS) in the Hebrew language. **החיסון ל-יחס כלפי** **COVID-19** .:דה רבה ,סמןאת התשובות שלך בסולם הדירוג ,בין 1 ל-5 2 = לא מסכים ,3 = ניטרלי ,4 = מסכים ו-5 = מסכים במידה רבה-.1 = לא מסכים במי.

FoCVV_1	-אני מפחד מאד מחיסון ל .1 COVID-19
FoCVV_2	-המחשבה על חיסון ל .2 COVID-19 גורמת לי לאי נוחות
FoCVV_3	-כפות הידיים שלי מזיעות כאשר שאני חושב על חיסון ל .3 COVID-19
FoCVV_4	-אני חושש לאבד את חיי בעקבות חיסון ל .4 COVID-19
FoCVV_5	-כשאני צופה בחדשות וסיפורים על חיסון ל .5 COVID-19 החברתיות, אני נהיה עצבני או חרד. ברשתות
FoCVV_6	-אני לא מצליח לישון כי אני מודאג מהתחסנות ל .6 COVID-19
FoCVV_7	-הלב שלי פועם בחוזקה או רועד כאשר אני חושב להתחסן ל .7 COVID-19

**Table A4:** Fear of Coronavirus Vaccination Scale (FoCVVS) in the Polish language. ***Postawa wobec przyjęcia szczepionki na COVID-19.*** Zaznacz swoje odpowiedzi na skali od 1 do 5, gdzie. 1 = zdecydowanie się NIE zgadzam, 2 = nie zgadzam się, 3 = mam neutralne nastawienie, 4 = zgadzam się, 5 = zdecydowanie zgadzam się.

FoCVV_1	Bardzo boję się szczepienia na koronawirusa.
FoCVV_2	Czuję się nieswojo, gdy myślę o szczepieniu na koronawirusa.
FoCVV_3	Moje dłonie stają się wilgotne, gdy myślę o szczepieniuna koronawirusa.
FoCVV_4	Boję się, że stracę życie z powodu szczepienia na koronawirusa.
FoCVV_5	Gdy oglądam wiadomości lub historie o szczepionce na koronawirusa w mediach społecznościowych, denerwuję się lub niepokoję.
FoCVV_6	Nie mogę spać, gdyż martwię się, że mogę otrzymać szczepionkę na koronawirusa.
FoCVV_7	Serce wali mi jak młotem, gdy myślę o zaszczepieniu na koronawirusa.

**Table A5:** Fear of Coronavirus Vaccination Scale (FoCVVS) in the Slovenian language. ***Odnos do cepiva proti COVID-19.*** Označite svoj odgovor v skladu z lestvico od 1 do 5: 1 = popolnoma se ne strinjam, 2 = se ne strinjam, 3 = nevtralno, 4 = se strinjam in 5 = popolnoma se strinjam.

FoCVV_1	Zelo se bojim cepiva proti COVID-19.
FoCVV_2	Ob razmišljanju na cepivo proti COVID-19 se počutim nelagodno.
FoCVV_3	Ko pomislim na cepivo proti COVID-19, se mi začnejo potiti dlani.
FoCVV_4	Bojim se, da lahko zaradi cepiva proti COVID-19 izgubim svoje življenje.
FoCVV_5	Ko gledam novice ali zgodbe o cepivu proti COVID-19 na socialnih omrežjih, postanem živčen/-a ali nemiren/-a.
FoCVV_6	Zaradi skrbi o cepivu proti COVID-19 ne morem spati.
FoCVV_7	Ko pomislim na to, da bi se cepil/-a proti koronavirusu, mi začne srce močno biti oz. poskakovati.

## References

[1] Haas E.J., Angulo F.J., McLaughlin J.M., Anis E., Singer S.S., Khan F., Brooks N., Smaja M., Mircus G., Pan K. (2021). Impact and effectiveness of mRNA BNT162b2 vaccine against SARS-CoV-2 infections and COVID-19 cases, hospitalisations, and deaths following a nationwide vaccination campaign in Israel: An observational study using national surveillance data. Lancet.

[2] Lopez Bernal J., Andrews N., Gower C., Robertson C., Stowe J., Tessier E., Simmons R., Cottrell S., Roberts R., O’Doherty M. (2021). Effectiveness of the Pfizer-BioNTech and Oxford-AstraZeneca vaccines on covid-19 related symptoms, hospital admissions, and mortality in older adults in England: Test negative case-control study. BMJ.

[3] Menni C., Klaser K., May A., Polidori L., Capdevila J., Louca P., Sudre C.H., Nguyen L.H., Drew D.A., Merino J. (2021). Vaccine side-effects and SARS-CoV-2 infection after vaccination in users of the COVID Symptom Study app in the UK: A prospective observational study. Lancet Infect. Dis..

[4] Agrawal V., Cantor J.H., Sood N., Whaley C.M. (2021). The Impact of the COVID-19 Vaccine Distribution on Mental Health Outcomes.

[5] Aw J., Seah S.S.Y., Seng B.J.J., Low L.L. (2022). COVID-19-related vaccine hesitancy among community hospitals’ healthcare workers in Singapore. Vaccines.

[6] Habib S.S., Alamri M.S., Alkhedr M.M., Alkhorijah M.A., Jabaan R.D., Alanzi M.K. (2022). Knowledge and attitudes of medical students toward COVID-19 vaccine in Saudi Arabia. Vaccines.

[7] Kowalski E., Stengel A., Schneider A., Goebel-Stengel M., Zipfel S., Graf J. (2022). How to motivate SARS-CoV-2 convalescents to receive a booster vaccination? Influence on vaccination willingness. Vaccines.

[8] Miyachi T., Sugano Y., Tanaka S., Hirayama J., Yamamoto F., Nomura K. (2022). COVID-19 vaccine intention and knowledge, literacy, and health beliefs among Japanese university students. Vaccines.

[9] Yoshida M., Kobashi Y., Kawamura T., Shimazu Y., Nishikawa Y., Omata F., Zhao T., Yamamoto C., Kaneko Y., Nakayama A. (2022). Factors associated with COVID-19 vaccine booster hesitancy: A retrospective cohort study, Fukushima vaccination community survey. Vaccines.

[10] Patwary M.M., Alam M.A., Bardhan M., Disha A.S., Haque M.Z., Billah S.M., Kabir M.P., Browning M.H.E.M., Rahman M.M., Parsa A.D. (2022). COVID-19 vaccine acceptance among low- and lower-middle-income countries: A rapid systematic review and meta-analysis. Vaccines.

[11] Patwary M.M., Bardhan M., Haque M.Z., Sultana R., Alam M.A., Browning M.H.E.M. (2022). COVID-19 vaccine acceptance rate and its factors among healthcare students: A systematic review with meta-analysis. Vaccines.

[12] Ritchie H., Mathieu E., Rodés-Guirao L., Appel C., Giattino C., Ortiz-Ospina E., Hasell J., Macdonald B., Beltekian D., Roser M. (2020). Coronavirus Pandemic (COVID-19). OurWorldInData.org. https://ourworldindata.org/coronavirus.

[13] Aron M.B., Connolly E., Vrkljan K., Zaniku H.R., Nyirongo R., Mailosi B., Ruderman T., Barnhart D.A., on behalf of the Partners In Health Cross-Site COVID-19 Cohort Research Network (2022). Attitudes toward COVID-19 vaccines among patients with complex non-communicable disease and their caregivers in rural Malawi. Vaccines.

[14] Gao X., Li H., He W., Zeng W. (2021). COVID-19 Vaccine Hesitancy Among Medical Students: The Next COVID-19 Challenge in Wuhan, China. Disaster Med. Public Health Prep..

[15] Galanis P., Vraka I., Katsiroumpa A., Siskou O., Konstantakopoulou O., Katsoulas T., Mariolis-Sapsakos T., Kaitelidou D. (2022). Predictors of willingness of the general public to receive a second COVID-19 booster dose or a new COVID-19 vaccine: A cross-sectional study in Greece. Vaccines.

[16] Pastorino R., Villani L., Mariani M., Ricciardi W., Graffigna G., Boccia S. (2021). Impact of COVID-19 Pandemic on Flu and COVID-19 Vaccination Intentions among University Students. Vaccines.

[17] Tucker J.S., D’Amico E.J., Pedersen E.R., Garvey R., Rodriguez A., Klein D.J. (2022). COVID-19 vaccination rates and attitudes among young adults with recent experiences of homelessness. J. Adolesc. Health.

[18] Wong C.H., Zhong C.C., Chung V.C., Nilsen P., Wong E.L., Yeoh E.-k. (2022). Barriers and facilitators to receiving the COVID-19 vaccination and development of theoretically-informed implementation strategies for the public: Qualitative study in Hong Kong. Vaccines.

[19] Zhu X.M., Yan W., Sun J., Liu L., Zhao Y.M., Zheng Y.B., Que J.Y., Sun S.W., Gong Y.M., Zeng N. (2022). Patterns and influencing factors of COVID-19 vaccination willingness among college students in China. Vaccine.

[20] Hamilton K., Hagger M.S. (2022). The Vaccination Concerns in COVID-19 Scale (VaCCS): Development and validation. PLoS ONE.

[21] Gregory M.E., MacEwan S.R., Powell J.R., Volney J., Kurth J.D., Kenah E., Panchal A.R., McAlearney A.S. (2022). The COVID-19 vaccine concerns scale: Development and validation of a new measure. Hum. Vaccines Immunother..

[22] Kotta I., Kalcza-Janosi K., Szabo K., Marschalko E.E. (2022). Development and Validation of the Multidimensional COVID-19 Vaccine Hesitancy Scale. Hum. Vaccines Immunother..

[23] Sato R., Fintan B. (2020). Fear, knowledge, and vaccination behaviors among women in Northern Nigeria. Hum. Vaccines Immunother..

[24] Malas O., Tolsá M.D. (2021). Vaccination Fear Scale (VFS-6): Development and Initial Validation. Mediterr. J. Clin. Psychol..

[25] Ahorsu D.K., Lin C.Y., Imani V., Saffari M., Griffiths M.D., Pakpour A.H. (2022). The Fear of COVID-19 Scale: Development and Initial Validation. Int. J. Ment. Health Addict..

[26] Elemo A.S., Satici S.A., Griffiths M.D. (2020). The Fear of COVID-19 Scale: Psychometric Properties of the Ethiopian Amharic Version. Int. J. Ment. Health Addict..

[27] Giordani R.C.F., Zanoni da Silva M., Muhl C., Giolo S.R. (2022). Fear of COVID-19 scale: Assessing fear of the coronavirus pandemic in Brazil. J. Health Psychol..

[28] Martínez-Lorca M., Martínez-Lorca A., Criado-Álvarez J.J., Armesilla M., Latorre J.M. (2020). The fear of COVID-19 scale: Validation in Spanish university students. Psychiatry Res..

[29] Caycho-Rodríguez T., Vilca L.W., Cervigni M., Gallegos M., Martino P., Portillo N., Barés I., Calandra M., Burgos Videla C. (2022). Fear of COVID-19 scale: Validity, reliability and factorial invariance in Argentina’s general population. Death Stud..

[30] Tzur Bitan D., Grossman-Giron A., Bloch Y., Mayer Y., Shiffman N., Mendlovic S. (2020). Fear of COVID-19 scale: Psychometric characteristics, reliability and validity in the Israeli population. Psychiatry Res..

[31] Masuyama A., Shinkawa H., Kubo T. (2022). Validation and psychometric properties of the Japanese version of the fear of COVID-19 scale among adolescents. Int. J. Ment. Health Addict..

[32] Alimoradi Z., Ohayon M.M., Griffiths M.D., Lin C.Y., Pakpour A.H. (2022). Fear of COVID-19 and its association with mental health-related factors: Systematic review and meta-analysis. BJPsych Open.

[33] Luo F., Ghanei Gheshlagh R., Dalvand S., Saedmoucheshi S., Li Q. (2021). Systematic Review and Meta-Analysis of Fear of COVID-19. Front. Psychol..

[34] Babicki M., Malchrzak W., Hans-Wytrychowska A., Mastalerz-Migas A. (2021). Impact of vaccination on the sense of security, the anxiety of COVID-19 and quality of life among Polish. A nationwide online survey in Poland. Vaccines.

[35] Alam M.D., Paul S.K., Momi M., Ni L., Xu Y. (2022). Factors associated with psychological outcomes among vaccinated and unvaccinated health care workers against COVID-19 infection in Bangladesh. Front. Med..

[36] Rogowska A.M., Chilicka K., Ochnik D., Paradowska M., Nowicka D., Bojarski D., Tomasiewicz M., Filipowicz Z., Grabarczyk M., Babińska Z. (2022). Network Analysis of Well-Being Dimensions in Vaccinated and Unvaccinated Samples of University Students from Poland during the Fourth Wave of the COVID-19 Pandemic. Vaccines.

[37] Reyna V.F. (2012). Risk perception and communication in vaccination decisions: A fuzzy-trace theory approach. Vaccine.

[38] Beaton D.E., Bombardier C., Guillemin F., Ferraz M.B. (2000). Guidelines for the Process of Cross-Cultural Adaptation of Self-Report Measures. Spine.

[39] Ochnik D., Rogowska A.M., Schütz A., Held M.J., Benatov J., Arzenšek A. Mental Health of Young Adults during COVID-19 Pandemic in Poland, Germany, Slovenia, and Israel: A Longitudinal Study. https://osf.io/4nh5m/.

[40] Pilch I., Kurasz Z., Turska-Kawa A. (2021). Experiencing fear during the pandemic: Validation of the fear of COVID-19 scale in Polish. PeerJ.

[41] DeSalvo K.B., Fisher W.P., Tran K., Bloser N., Merrill W., Peabody J. (2006). Assessing measurement properties of two single-item general health measures. Qual. Life Res..

[42] Diener E., Emmons R.A., Larsen R., Griffin S. (1985). The satisfaction with life scale. J. Personal. Assess..

[43] Cohen S., Kamarck T., Mermelstein R. (1983). A global measure of perceived stress. J. Health Soc. Behav..

[44] Spitzer R.L., Kroenke K., Williams J.B., Löwe B. (2006). A brief measure for assessing generalized anxiety disorder: The GAD-7. Arch. Intern. Med..

[45] Kroenke K., Spitzer R.L., Williams J.B. (2001). The PHQ-9: Validity of a brief depression severity measure. J. Gen. Intern. Med..

[46] JASP Team (2020). 2020 JASP.

[47] (2019). IBM SPSS Statistics for Windows.

[48] Gaskin J., Lim J. (2016). Master Validity Tool [AMOS Plugin]. http://statwiki.gaskination.com/index.php?title=Main_Page.

[49] Field A. (2005). Discovering Statistics Using SPSS.

[50] Hair J., Tatham R., Anderson R., Black W. (2006). Multivariate Data Analysis.

[51] Fornell C.G., Larcker D.F. (1981). Evaluating structural equation models with unobservable variables and measurement error. J. Market. Res..

[52] Henseler J., Ringle C.M., Sarstedt M. (2015). A new criterion for assessing discriminant validity in variance-based structural equation modeling. J. Acad. Mark. Sci..

[53] Kline R.B. (2011). Principles and Practice of Structural Equation Modeling.

[54] Hu L.-T., Bentler P.M. (1999). Cutoff criteria for fit indexes in covariance structure analysis: Conventional criteria versus new alternatives. Struct. Equ. Model..

[55] Chen F.F. (2007). Sensitivity of Goodness of Fit Indexes to Lack of Measurement Invariance. Struct. Equ. Model..

[56] Gregorich S.E. (2006). Do self-report instruments allow meaningful comparisons across diverse population groups? Testing measurement invariance using the confirmatory factor analysis framework. Med. Care.

[57] Ochnik D., Rogowska A.M., Arzenšek A., Benatov J. (2022). Can Fear of COVID-19 Be Predicted by Religiosity and Trust in Institutions among Young Adults? A Prospective Cross-National Study. Int. J. Environ. Res. Public Health.

[58] Pandey K., Thurman M., Johnson S.D., Acharya A., Johnston M., Klug E.A., Olwenyi O.A., Rajaiah R., Byrareddy S.N. (2021). Mental Health Issues During and After COVID-19 Vaccine Era. Brain Res. Bull..

[59] Alzubaidi H., Samorinha C., Saddik B., Saidawi W., Abduelkarem A.R., Abu-Gharbieh E., Sherman S.M. (2021). A mixed-methods study to assess COVID-19 vaccination acceptability among university students in the United Arab Emirates. Hum. Vaccines Immunother..

[60] Lo Moro G., Cugudda E., Bert F., Raco I., Siliquini R. (2022). Vaccine Hesitancy and Fear of COVID-19 Among Italian Medical Students: A Cross-Sectional Study. J. Commun. Health.

