# Supplementary material for: Adaptation and Preliminary Validation of the Fear of Coronavirus Vaccination Scale in the Prospective Study among a Representative Sample of Polish, Israeli, Slovenian, and German Adults during the COVID-19 Pandemic
Source: Int J Environ Res Public Health. 2022 Sep 14;19(18):11587. doi: 10.3390/ijerph191811587 (PMC9517357; doi:10.3390/ijerph191811587)
Supplement: Supplementary file 1 [file ijerph-19-11587-s001.zip › ijerph-1881935-supplementary.pdf]

Adaptation and Preliminary Validation of the Fear of Coronavirus Vaccination Scale in the prospective study among a representative sample of German, Israel, Polish and Slovenian adults during the COVID-19 pandemic

(D.Ochnik, A.M.Rogowska, J.Benatov, A.Arzenšek)

Supplementary Material

Table S1. Descriptive statistics for the sample of adults from Germany (*n* = 418)

Item	Range	*M*	*SD*	*Mdn*	*Skew.*	*Kurt.*	1	2	3	4	5	6
1. FoCVV_1	1-5	2.76	1.19	3	–0.01	–0.91						
2. FoCVV_2	1-5	2.90	1.18	3	–0.07	–0.83	0.72***					
3. FoCVV_3	1-5	2.21	1.20	2	0.62	–0.63	0.54***	0.51***				
4. FoCVV_4	1-5	2.55	1.32	3	0.29	–1.07	0.68***	0.55***	0.65***			
5. FoCVV_5	1-5	2.44	1.20	2	0.31	–0.87	0.60***	0.52***	0.68***	0.68***		
6. FoCVV_6	1-5	2.14	1.23	2	0.67	–0.71	0.51***	0.43***	0.73***	0.65***	0.68***	
7. FoCVV_7	1-5	2.14	1.20	2	0.63	–0.74	0.53***	0.43***	0.75***	0.64***	0.71***	0.86***

Table S2. Descriptive statistics for the sample of adults from Israel (*n* = 428)

Item	Range	*M*	*SD*	*Mdn*	*Skew.*	*Kurt.*	1	2	3	4	5	6
1. FoCVV_1	1-5	2.52	1.34	2	0.39	–1.05						
2. FoCVV_2	1-5	2.51	1.37	2	0.41	–1.07	0.83***					
3. FoCVV_3	1-5	1.61	0.97	1	1.62	1.94	0.50***	0.51***				
4. FoCVV_4	1-5	1.97	1.23	1	1.05	–0.05	0.64***	0.65***	0.63***			
5. FoCVV_5	1-5	2.28	1.30	2	0.6	–0.89	0.64***	0.68***	0.57***	0.69***		
6. FoCVV_6	1-5	1.64	0.96	1	1.5	1.56	0.50***	0.52***	0.77***	0.66***	0.63***	
7. FoCVV_7	1-5	1.65	0.99	1	1.46	1.26	0.51***	0.52***	0.79***	0.64***	0.63***	0.83***

Table S3. Descriptive statistics for the sample of adults from Poland (*n* = 446)

Item	Range	*M*	*SD*	*Mdn*	*Skew.*	*Kurt.*	1	2	3	4	5	6
1. FoCVV_1	1-5	3.06	1.23	3	–0.11	–0.79						
2. FoCVV_2	1-5	3.11	1.25	3	–0.19	–0.88	0.76***					
3. FoCVV_3	1-5	2.39	1.25	2	0.47	–0.79	0.57***	0.56***				
4. FoCVV_4	1-5	2.82	1.33	3	0.12	–1.07	0.65***	0.70***	0.60***			
5. FoCVV_5	1-5	2.91	1.25	3	–0.06	–0.94	0.67***	0.63***	0.58***	0.70***		
6. FoCVV_6	1-5	2.33	1.19	2	0.43	–0.82	0.49***	0.47***	0.75***	0.59***	0.58***	
7. FoCVV_7	1-5	2.29	1.18	2	0.46	–0.76	0.49***	0.50***	0.77***	0.62***	0.56***	0.87***

Table S4. Descriptive statistics for the sample of adults from Slovenia (*n* = 431)

Item	Range	*M*	*SD*	*Mdn*	*Skew.*	*Kurt.*	1	2	3	4	5	6
1. FoCVV_1	1-5	2.90	1.25	3	0.10	–0.84						
2. FoCVV_2	1-5	3.13	1.30	3	–0.11	–1.02	0.74***					
3. FoCVV_3	1-5	2.02	1.20	2	1.03	0.16	0.56***	0.48***				
4. FoCVV_4	1-5	2.52	1.31	2	0.46	–0.87	0.62***	0.63***	0.65***			
5. FoCVV_5	1-5	2.73	1.27	3	0.17	–0.97	0.47***	0.52***	0.51***	0.57***		
6. FoCVV_6	1-5	1.69	1.00	2	1.51	1.82	0.41***	0.36***	0.73***	0.51***	0.48***	
7. FoCVV_7	1-5	1.85	1.10	2	1.27	0.89	0.50***	0.45***	0.78***	0.59***	0.53***	0.82***

Note. FoCVV = fear of Coronavirus vaccination; *M* = mean score; *SD* = standard deviation; *Mdn* = median; Skew. = skewness; Kurt. = kurtosis; ***$p < 0.001$.

Adaptation and Preliminary Validation of the Fear of Coronavirus Vaccination Scale in the prospective study among a representative sample of German, Israel, Polish and Slovenian adults during the COVID-19 pandemic

(D.Ochnik, A.M.Rogowska, J.Benatov, A.Arzenšek)

Table S5. One-way ANOVA results for the Fear of Coronavirus Vaccination Scale across four languages

Variable	Polish (1) (*n* = 446)		German (2) (*n* = 418)		Slovenian (3) (*n* = 431)		Hebrew (4) (*n* = 428)		*F* (3, 1719)	*p*	η_p^2	Bonferroni posthoc test of differences
	M	*SD*	*M*	*SD*	*M*	*SD*	*M*	*SD*				
FoCVV_1	2.74	1.24	2.68	1.29	2.81	1.25	2.22	1.29	19.19	< 0.001	0.032	1/4, 2/4, 3/4
FoCVV_2	2.85	1.28	2.74	1.27	2.96	1.31	2.26	1.29	25.16	< 0.001	0.042	1/4, 2/4, 3/4
FoCVV_3	2.19	1.18	2.13	1.23	1.97	1.12	1.55	0.90	29.36	< 0.001	0.049	1/3, 1/4, 2/4, 3/4
FoCVV_4	2.60	1.32	2.46	1.32	2.65	1.34	1.85	1.14	35.60	< 0.001	0.058	1/4, 2/4, 3/4
FoCVV_5	2.59	1.23	2.39	1.24	2.79	1.27	2.01	1.20	30.85	< 0.001	0.051	1/4, 2/3, 2/4, 3/4
FoCVV_6	2.23	1.20	2.06	1.17	1.74	1.00	1.54	0.87	37.22	< 0.001	0.061	1/3, 1/4, 2/3, 2/4, 3/4
FoCVV_7	2.19	1.18	2.14	1.21	1.91	1.14	1.60	0.98	24.20	< 0.001	0.041	1/3, 1/4, 2/3, 2/4, 3/4
Emotional	10.79	4.55	10.27	4.40	11.21	4.36	8.34	4.34	35.52	< 0.001	0.058	1/4, 2/3, 2/4, 3/4
Physiological	6.61	3.36	6.33	3.38	5.62	2.97	4.69	2.55	33.49	< 0.001	0.055	1/3, 1/4, 2/3, 2/4, 3/4
Total	17.40	7.41	16.60	7.35	16.83	6.70	13.02	6.48	34.79	< 0.001	0.057	1/4, 2/4, ¾

Note. FoCVV = fear of coronavirus vaccination.

Table S6. The regression loadings in Model 3 across four languages

Factor	Variable	German (*n* = 418)		Hebrew (*n* = 428)		Polish (*n* = 446)		Slovenian (*n* = 431)	
		B (95% CI)	β	*B* (95% CI)	β	*B* (95% CI)	β	*B* (95% CI)	β
Emotional	FoCVV_1	0.31 (0.21-0.39)	0.71	0.30 (0.23-0.37)	0.67	0.37 (0.30-0.45)	0.73	0.37 (0.27-0.47)	0.72
	FoCVV_2	0.25 (0.16-0.32)	0.59	0.33 (0.25-0.40)	0.72	0.41 (0.33-0.48)	0.78	0.41 (0.29-0.52)	0.75
	FoCVV_4	0.37 (0.26-0.45)	0.83	0.35 (0.28-0.41)	0.88	0.45 (0.37-0.52)	0.83	0.47 (0.34-0.58)	0.84
	FoCVV_5	0.38 (0.26-0.47)	0.91	0.37 (0.30-0.44)	0.88	0.45 (0.38-0.52)	0.90	0.35 (0.25-0.43)	0.67
Physiological	FoCVV_3	0.38 (0.27-0.49)	0.93	0.27 (0.21-0.33)	0.84	0.45 (0.36-0.53)	0.94	0.41 (0.30-0.52)	0.89
	FoCVV_6	0.36 (0.27-0.44)	0.92	0.27 (0.21-0.33)	0.88	0.46 (0.38-0.53)	0.95	0.29 (0.20-0.36)	0.71
	FoCVV_7	0.38 (0.28-0.47)	0.94	0.34 (0.27-0.41)	0.96	0.45 (0.37-0.52)	0.93	0.40 (0.29-0.49)	0.85
Total	Emotional	2.80 (2.22-3.94)	0.94	2.64 (2.22-3.30)	0.94	2.22 (1.89-2.74)	0.91	2.19 (2.19-1.75)	0.91
	Physiological	2.83 (2.23-4.00)	0.94	2.61 (2.07-3.33)	0.93	2.26 (1.93-2.78)	0.91	2.20 (1.64-3.12)	0.91

Note. FoCVV = fear of Coronavirus vaccination; *B* = unstandardized estimate; β = standardized estimate; CI = confidence interval.

Adaptation and Preliminary Validation of the Fear of Coronavirus Vaccination Scale in the prospective study among a representative sample of German, Israel, Polish and Slovenian adults during the COVID-19 pandemic

(D.Ochnik, A.M.Rogowska, J.Benatov, A.Arzenšek)

Table S7. Differences in fear of coronavirus vaccination and well-being dimensions between unvaccinated and vaccinated participants during T1 of the third wave of the COVID-19 pandemic

Variable	Unvaccinated (n = 1390)		*Vaccinated* (n = 333)		t(1721)	p
	M	*SD*	*M*	*SD*		
Fear of coronavirus vaccination scale (FoCVVS)	17.58	6.97	13.47	6.69	9.73	< 0.001
FoCVVS Emotional symptoms	11.37	4.37	8.38	4.04	11.42	< 0.001
FoCVVS Physiological symptoms	6.21	3.26	5.10	3.00	5.66	< 0.001
Fear of COVID-10 Scale (FoCV-19S)	16.01	6.51	16.40	6.66	–0.96	0.336
FoCV-19S Emotional symptoms	10.21	3.96	10.74	4.16	–2.17	0.030
FoCV-19S Physiological symptoms	5.81	3.05	5.66	3.23	0.77	0.439
Physical health	5.83	1.86	6.81	1.89	–8.60	< 0.001
Life satisfaction	20.84	5.75	21.26	6.41	–1.18	0.238
Perceived stress	19.82	6.56	19.02	6.67	2.00	0.046
Anxiety	7.43	5.64	6.08	5.38	3.78	< 0.001
Depression	8.08	6.78	7.69	6.81	0.95	0.341

Table S8. Differences in fear of vaccination and well-being dimensions between unvaccinated and vaccinated participants during the T2 of the third wave of the COVID-19 pandemic

Variable	Unvaccinated (n = 1390)		*Vaccinated* (n = 333)		t(1721)	p
	M	*SD*	*M*	*SD*		
Fear of coronavirus vaccination scale (FoCVVS)	11.01	7.00	5.97	6.39	15.18	< 0.001
FoCVVS Emotional symptoms	7.62	4.34	4.00	3.94	17.59	< 0.001
FoCVVS Physiological symptoms	3.39	3.29	1.97	2.78	9.37	< 0.001
Fear of COVID-10 Scale (FoCV-19S)	8.30	6.46	7.87	6.63	1.35	0.179
FoCV-19S Emotional symptoms	5.63	3.93	5.44	4.05	0.95	0.345
FoCV-19S Physiological symptoms	2.67	2.94	2.42	3.03	1.69	0.092
Physical health	5.70	1.82	6.33	1.99	–6.82	< 0.001
Life satisfaction	21.05	5.99	21.27	6.31	–0.73	0.464
Perceived stress	17.93	5.65	17.49	6.08	1.53	0.125
Anxiety	6.80	5.71	6.63	5.67	0.60	0.548
Depression	7.89	6.97	7.81	6.86	0.26	0.795

Adaptation and Preliminary Validation of the Fear of Coronavirus Vaccination Scale in the prospective study among a representative sample of German, Israel, Polish and Slovenian adults during the COVID-19 pandemic

(D.Ochnik, A.M.Rogowska, J.Benatov, A.Arzenšek)

Table S9. Pearson's correlations between three scales of fear of vaccination (general, emotional, and physiological) during T2 and well-being dimensions at T1, stratified into the total sample of vaccinated and unvaccinated participants

**Variables at T1**	**Fear of coronavirus vaccination T2**		**Emotional T2**		**Physiological T2**	
	r	**95% CI (LL, UL)**	***r***	**95% CI (LL, UL)**	***r***	**95% CI (LL, UL)**
FoCVVS Total	0.66***	(0.64, 0.69)	0.64***	(0.61, 0.66)	0.60***	(0.57, 0.63)
FoCVVS Emotional	0.62***	(0.59, 0.65)	0.64***	(0.62, 0.67)	0.50***	(0.46, 0.53)
FoCVVS Physiological	0.60***	(0.57, 0.63)	0.51***	(0.47, 0.54)	0.63***	(0.60, 0.65)
FoCV-19S Total	0.39***	(0.35, 0.43)	0.32***	(0.28, 0.36)	0.42***	(0.38, 0.46)
FoCV-19S Emotional	0.31***	(0.27, 0.35)	0.28***	(0.24, 0.32)	0.31***	(0.27, 0.35)
FoCV-19S Physiological	0.42***	(0.38, 0.45)	0.32***	(0.27, 0.36)	0.49***	(0.45, 0.52)
Physical health (GSRH)	–0.04	(–0.08, 0.01)	–0.06**	(–0.11, –0.02)	–0.01	(–0.05, 0.04)
Life satisfaction (SWLS)	–0.03	(–0.08, 0.02)	–0.03	(–0.08, 0.01)	–0.01	(–0.06, 0.03)
Stress (PSS-10)	0.21***	(0.17, 0.26)	0.21***	(0.16, 0.25)	0.18***	(0.13, 0.23)
Anxiety (GAD-7)	0.35***	(0.31, 0.39)	0.29***	(0.34, 0.25)	0.37***	(0.33, 0.41)
Depression (PHQ-9)	0.27***	(0.32, 0.40)	0.22***	(0.18, 0.27)	0.29***	(0.24, 0.33)

Note. FoCVVS = Fear of Coronavirus Vaccination Scale; FoCV-19S = Fear of COVID-19 Scale; GSRH = General Self-Rated Health; SWLS = Satisfaction With Life Scale; PSS-10 = 10-item Perceived Stress Scale; GAD-7 = 7-item Generalized Anxiety Disorder; PHQ-9 = 9-item Patient Health Questionnaire; CI = confidence interval; LL = lower level; UL = upper level. **$p < 0.01$, ***$p < 0.001$
